# Evaluation of six CTLA-4 polymorphisms in high-risk melanoma patients receiving adjuvant interferon therapy in the He13A/98 multicenter trial

**DOI:** 10.1186/1479-5876-8-108

**Published:** 2010-11-03

**Authors:** Helen Gogas, Urania Dafni, Henry Koon, Maria Spyropoulou-Vlachou, Yannis Metaxas, Elizabeth Buchbinder, Eirini Pectasides, Dimosthenis Tsoutsos, Aristidis Polyzos, Alexandros Stratigos, Christos Markopoulos, Petros Panagiotou, George Fountzilas, Ourania Castana, Pantelis Skarlos, Michael B Atkins, John M Kirkwood

**Affiliations:** 1 First Department of Medicine, University of Athens, Medical School, Athens, Greece; 2 Laboratory of Biostatistics, University of Athens School of Nursing, Athens, Greece; 3 University Hospital Case Medical Center, Case Comprehensive Cancer Center, Cleveland, OH, USA; 4 Department of Immunology, National Tissue Typing Center, General Hospital of Athens, Greece; 5 Beth Israel Deaconess Medical Center, Harvard Medical School, Boston, Massachusetts, USA; 6 Department of Plastic Surgery and Microsurgery, G. Gennimatas General Hospital of Athens, Greece; 7 Department of Dermatology, University of Athens, "Andreas Sygros" Hospital, Athens, Greece; 8 Department of Medical Oncology, Papageorgiou Hospital, Aristotle University of Thessaloniki, School of Medicine, Thessaloniki, Greece; 9 Department of Plastic Surgery, Evagelismos Hospital, Athens, Greece; 10 Hellenic Cooperative Oncology Group, Data Office, Athens, Greece; 11 University of Pittsburgh Cancer Institute, Hillman Cancer Center, Pittsburgh, Pennsylvania, USA

## Abstract

**Purpose:**

Interferon is approved for adjuvant treatment of patients with stage IIb/III melanoma. The toxicity and uncertainty regarding survival benefits of interferon have qualified its acceptance, despite significant durable relapse prevention in a fraction of patients. Predictive biomarkers that would enable selection of patients for therapy would have a large impact upon clinical practice. Specific CTLA-4 polymorphisms have previously shown an association with response to CTLA-4 blockade in patients with metastatic melanoma and the development of autoimmunity.

**Experimental design:**

286 melanoma patients and 288 healthy controls were genotyped for six CTLA-4 polymorphisms previously suggested to be important (AG 49, CT 318, CT 60, JO 27, JO30 and JO 31). Specific allele frequencies were compared between the healthy and patient populations, as well as presence or absence of these in relation to recurrence. Alleles related to autoimmune disease were also investigated.

**Results:**

No significant differences were found between the distributions of CTLA-4 polymorphisms in the melanoma population compared with healthy controls. Relapse free survival (RFS) and overall survival (OS) did not differ significantly between patients with the alleles represented by these polymorphisms. No correlation between autoimmunity and specific alleles was shown. The six polymorphisms evaluated where strongly associated (Fisher's exact p-values < 0.001 for all associations) and significant linkage disequilibrium among these was indicated.

**Conclusion:**

No polymorphisms of CTLA-4 defined by the SNPs studied were correlated with improved RFS, OS, or autoimmunity in this high-risk group of melanoma patients.

## Introduction

Interferon alfa (IFNα) was the first cytokine to demonstrate antitumor activity in patients with advanced melanoma and has been widely tested as adjuvant therapy in patients at intermediate and high risk of melanoma recurrence and associated mortality. Adjuvant treatment of patients with stage IIB/III melanoma with high-dose IFNα (HDI)was approved by the United States Food and Drug Administration (FDA) in 1995, and subsequently by regulatory authorities worldwide [1]. Despite the ability of this regimen to reduce relapse and mortality by up to 33% [2] the tolerability of this regimen has been an issue, due to the frequent occurrence of flu-like symptoms, including fatigue and anorexia, as well as hepatic abnormalities and occasional depression.

Attempts to identify the subset of patients destined to benefit from adjuvant treatment with IFNα-2b have failed to discover clinical or demographic features of the patient population most likely benefit from HDI therapy Correlative studies have been undertaken over the years, demonstrating a variety of immunological responses subsequent to therapy [3,4]. There is a critical need for greater understanding of the immunological and disease-related variables that predict clinical benefit from IFNα-2b. The identification of predictive markers would permit selection of patients likely to benefit and would enable the 66% of patients unlikely to benefit to avoid the attendant toxicity. The immunotherapies that benefit advanced melanoma include IL-2, which has also been shown to induce autoimmune reactions, thyroiditis, and vitiligo. [5-14], A variety of autoimmune phenomena have been reported to occur during adjuvant therapy with HDI. In a substudy of a large randomized trial of HDI in patients with stage IIB/III melanoma, 26% of 200 patients developed antithyroid antibodies or other autoimmune manifestations [15]. The appearance of autoantibodies or clinical manifestations of autoimmunity was associated with significant improvements in relapse-free (RFS) and overall survival (OS) (p < .001). This suggested that the induction of autoimmunity could be a surrogate marker for interferon efficacy. However, as autoimmunity was observed only after a median of three months --and in some instances, more than a year from the start of IFNα-2b therapy, the development of autoimmunity *per se *could not serve as a criterion for selecting patients to initiate therapy.

The human CTLA-4 gene is located on chromosome 2q33, in a region that is associated with susceptibility for autoimmune disease [16]. Multiple polymorphisms within the CTLA-4 gene have been found to be associated with susceptibility to autoimmune diseases (e.g., the GG allele of the +49 AG polymorphism is associated with decreased expression of CTLA-4 upon T-cell activation and thus a higher proliferation of T-cells) [17-20]. Additionally, in a phase I study of 19 patients receiving anti-CTLA-4 monoclonal antibody with multiple melanoma peptides and Montanide ISA 51, three of four (75%) patients with the CTLA-4 allele JO 30 (GG) developed autoimmune symptoms, and only two (50%) experienced disease relapse. Of the remaining 15 patients expressing either the AA or AG alleles, only five (33%) developed autoimmune symptoms and 10 (67%) experienced disease relapse [21].

We therefore evaluated six CTLA-4 *S*ingle *N*ucleotide *P*olymorphisms (SNPs) in a cohort of high-risk melanoma patients enrolled in a study of two regimens of HDI, and compared the distribution of these SNPS to those found in healthy controls (healthy unrelated individuals from the Donor Marrow Registry of the National Tissue Typing Center, Athens, Greece). The correlation of the CTLA-4 polymorphisms associated with the development of autoimmune diseases and the HLA Cw*06 allele which predisposes to psoriasis was also studied as a consequence of our observation that this allele was associated with the disease outcome and induction of autoimmunity in patients treated with adjuvant HDI [22].

## Materials and Methods

### Materials

We genotyped DNA isolated from the peripheral blood of a total of 286 patients with melanoma and a panel of 288 randomly selected healthy unrelated Greek individuals that served as a control population, for 6 CTLA4-SNPs, namely CT 60, AG 49, CT 318 , JO 27, JO 30 and JO 31. CT 318 is located within the promoter region of the CTLA-4 gene, A/G49 is located at exon 1, while the rest of the SNPs tested are located at the 3' untranslated region of CTLA-4.

Patients participating in this study were enrolled in Trial 13A/98, a prospective, multicenter, randomized phase III trial conducted at 13 institutions by the Hellenic Cooperative Oncology Group (HeCOG). This trial, enrolled 364 patients with histologically documented AJCC stage IIB, IIC, or III primary cutaneous melanoma between 1998 and 2004. For patients with clinically uninvolved lymph nodes, stage was defined pathologically using sentinel lymph node (SLN) biopsy. Any patient with a positive SLN was required to undergo completion lymphadenectomy. All patients were assigned at random to receive one of the two treatment regimens within 2 months of initial surgery or 1.5 months of therapeutic lymph node dissection. The regimens used were a modification of the E1684 regimen [23]. Group A patients received IFN-α2b (15 MIU/m^2^/day IV 5 days per week for 4 weeks) followed by observation. Group B patients received the same induction dose for 4 weeks followed by subcutaneous therapy (10 MIU/day TIW) for an additional 48 weeks. The primary endpoints for the core protocol were RFS and OS by treatment group.

The CTLA-4 polymorphism sub-study reported here was conducted retrospectively in four institutions that had participated in the core protocol. This substudy had separate IRB approval, and all patients had provided written informed consent for provision of biological material for such future research studies at initiation of treatment. Blood samples for evaluation of CTLA-4 were drawn prior to treatment at the same time as samples for routine initial visit blood tests. The first 10 mL of blood collected was used for standard biochemistry and blood cell counts, and the second 3 mL was used for CTLA-4 testing.

The clinical outcome of patients was prospectively followed using standardized testing. Clinical staging consisted of medical history, physical exams, blood cell counts, blood biochemistry at 3-month intervals, and chest x-ray and liver ultrasound at 6-month intervals.

### Methods

DNA was isolated using the GenoPrep extraction system (GenoVision, Oslo, Norway) and the SNP-PCR was carried out with the following primers: CT 318 forward ACCCTTGTACTCCAGGAAATTCTC, reverse biotinylated-GGTTTAGCTGTTACGTCGAAAAGA, AG 49 forward TTTCAGCGGCACAAGGCTC, reverse biotinylated-GAGTGCAGGGCCAGGTCC, CT 60 forward GCAAGTCATTCTTGGAAGGTATC, reverse biotinylated-TGCCAATTGATTTATAAAGGACTG, JO 27 forward GAGCTGGTCAGCCGAGAT, reverse biotinylated- TGACACCACCCCTCCATAAT, JO 30 forward CAAAGCAAAACGCTGCCAATAA, reverse biotinylated- TCCAGTGGCAATAGGAGCTTTC, JO 31 forward TTGTCATGTTAGCCGTGCAGC, reverse biotinylated- CCACCACCACACCCAGGTAA. 50 ng of DNA were amplified in a 50 μL reaction containing 25 μL MasterMix (Illustra HotStart MasterMix, GE Healthcare, Buckinghamshire, UK) 1 μL (10 pmol) of each primer and denaturized water. PCR conditions were as follows: first, a 5 minute incubation at 95°C was performed, followed by 45 cycles of a 15 seconds denaturation step at 95°C, 30 seconds annealing step at 56°C and 15 seconds extension step at 72°C. There was a final extension step at 72°C for 5 minutes. We then genotyped the amplicons using Pyrosequencing technology (Biotage, Uppsala, Sweden). The PCR strand which was labeled by the biotinylated primer was captured on Streptavidin Sepharose™ High Performance beads (GE Healthcare, Uppsala, Sweden) and washed for 10 seconds in 70% ethanol to remove PCR residuals. Single-stranded DNAs were prepared after denaturation for 10 seconds with Denaturation Solution (Biotage, Uppsala, Sweden) and then they were treated for 5 seconds with appropriate Washing Buffer (Biotage, Uppsala, Sweden). Hybridization of sequencing primers to respective templates was carried out according to the standard protocol described by the manufacturer (Biotage, Uppsala, Sweden). All of the sequencing reactions were performed on the PyroMark™ ID pyrosequencer, using the PSQ 96 SNP Reagent Kit (Biotage AB) and analysis was done with PyroMark™ ID 1.0 software. The sequencing primers used were: 318C/T CACTTAGTTATCCAGATCCT, AG 49 GCTCAGCTGAACCTG, CT 60 TCACCACTATTTGGGATAT, JO 27 TACCAGAAGTTGAAGTGTAG, JO 30 TCTGTCAGCAAAGCC, and JO 31 ACCTCTTGAGGTCAGGAGT http://hapmap.ncbi.nlm.nih.gov/index.html.en.

### Statistical Analysis

Allele frequencies were defined as follows: Each individual was used as a unit and a particular allele was noted as present if detected in an individual. Specific allele frequencies were calculated both for the patient population and the healthy control population. Fisher's exact test was used for comparing the frequency of specific alleles (one observation per patient) between the healthy and patient populations as well as the frequency of recurrence between the population where the specific allele was present versus the population it was absent.

In addition, recurrence and specific allele frequencies were compared between patients with and without autoimmune responses as well as HLA-Cw*06Survival was evaluated from the date protocol treatment was started to the date of last follow-up or date of death from any cause. RFS was calculated from the initiation of treatment to the date on which relapse was first documented or on which death without documented relapse occurred. The Kaplan-Meier method was used for the estimation of RFS and OS curves. The reverse censoring method was used for calculating descriptive statistics for the follow-up time [24].

Cox regression analyses on RFS and OS were performed, evaluating the association of outcome to the presence of polymorphisms of CTLA-4 (AG 49, CT 60, CT 318, JO 27, JO 30, JO 31), as well as of the most frequent haplotypes. The combined effects of HLA-Cw*06, AG 49 and the presence of autoimmunity on RFS and OS were explored through a multivariate Cox model.

Maximum likelihood estimates of haplotype frequencies given a multilocus sample of genetic marker genotypes [3 different genotypes of the 6 polymorphisms] were generated using the expectation-maximization (EM) algorithm under the assumption of Hardy-Weinberg equilibrium (HWE). Linkage disequilibrium was explored for each pair of the 6 polymorphisms (PROC HAPLOTYPE). SAS 9.1 (SAS Institute Inc., Cary, NC, USA), was used for the statistical analysis.

## Results

The frequency patterns of CTLA-4 alleles were first evaluated in the healthy control and melanoma populations. There were no statistical differences in the incidence of CTLA-4 polymorphisms between melanoma patients and healthy controls (Table 1), except for JO 31, where the T/T allele was higher in controls (33.3% vs 24.3%) while G/G and G/T was lower (p = 0.047).

**Table 1:** Frequencies of CTLA-4 polymorphisms in melanoma patients and healthy controls

	Controls		Melanomas		
	**Number (N = 288)**	**%**	**Number (N = 286)**	**%**	**P**

**AG 49**					

A/A	152	52.8	132	46.2	0.27

A/G	111	38.5	128	44.8	

G/G	25	8.7	26	9.1	

**CT 318**					

C/C	230	79.9	229	80.1	0.94

C/T	57	19.8	55	19.2	

T/T	1	0.4	2	0.7	

**CT 60**					

A/A	90	31.3	65	22.7	0.071

A/G	135	46.9	151	52.8	

G/G	63	21.9	70	24.5	

**JO 27**					

C/C	90	31.3	73	25.5	0.32

C/T	143	49.7	153	53.5	

T/T	55	19.1	60	21.0	

**JO 30**					

A/A	95	33.0	72	25.2	0.12

A/G	138	47.9	151	52.8	

G/G	55	19.1	63	22.0	

**JO 31**					

T/T	96	33.3	71	24.8	0.047

G/T	144	50.0	151	52.8	

G/G	48	16.8	64	22.4	

**Table 2:** Univariate Cox Regression Models of Relapse-free Survival and Overall Survival

	No of events/No of patients	Median Relapse- free Survival (months)	P value	No of events/No of patients	Median Overall Survival (months)	P value
**AG 49**						

A/A	71/132	59.56	0.55	47/132	NR*	0.55

A/G	70/128	46.42		47/128	84.20	

G/G	17/26	35.35		11/26	63.38	

**CT 318**						

C/C	122/229	54.67	0.52	82/229	NR	0.36

C/T	34/55	47.67		21/55	NR	

T/T	2/2	37.35		2/2	51.02	

**CT 60**						
A/A	34/65	58.87	0.68	23/65	NR	0.64

A/G	85/151	47.67		54/151	84.20	

G/G	39/70	53.22		28/70	76.68	

**JO 27**						

C/C	37/73	58.87	0.60	26/73	NR	0.76

T/C	84/153	54.67		54/153	84.20	

T/T	37/60	37.29		25/60	76.68	

**JO 30**						

A/A	37/72	56.71	0.65	27/72	NR	0.74

G/A	83/151	54.80		52/151	NR	

G/G	38/63	37.29		26/63	76.68	

**JO 31**						

T/T	35/71	72.08	0.37	23/71	NR	0.50

G/T	85/151	47.67		56/151	80.69	

G/G	38/64	39.43		26/64	76.68	

* NR: Not Reached

Patient demographics and baseline characteristics have been described elsewhere [15,23]. With a median follow up of 70.7 months [only among patients alive (censored values), range 7.1-138.7 months], there were 158 recurrences (median RFS 55 months, range 1 to 115 months) and 105 deaths (median OS not reached yet, range 2 to 86 months).

**Figure 1:**
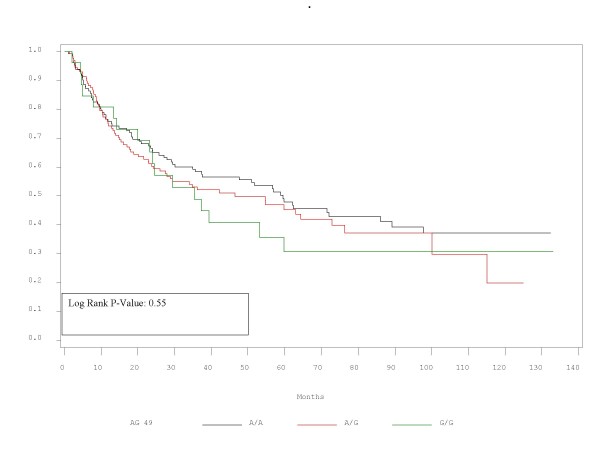
**RFS plot by A/G 49 status**.

**Figure 2:**
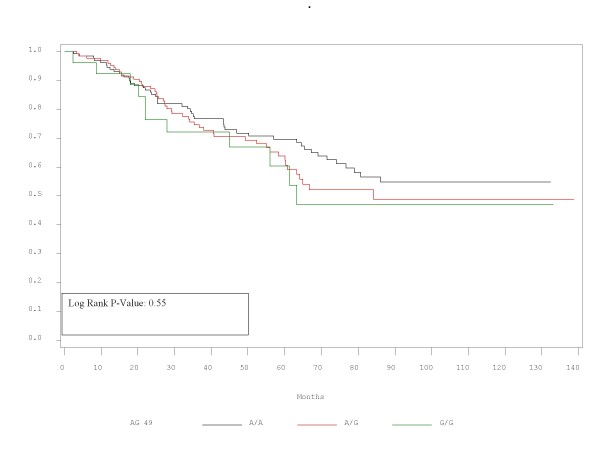
OS plot by A/G 49 status

**Figure 3:**
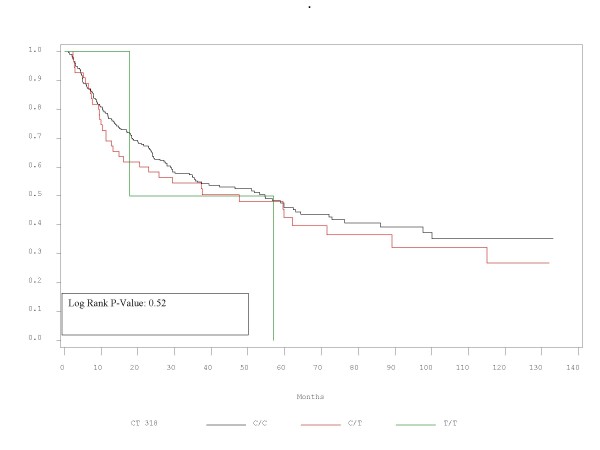
RFS plot by C/T 318 status

**Figure 4:**
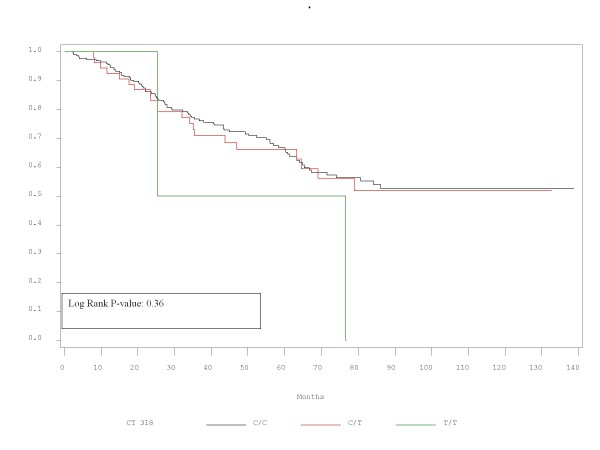
OS plot by C/T 318 status

**Figure 5:**
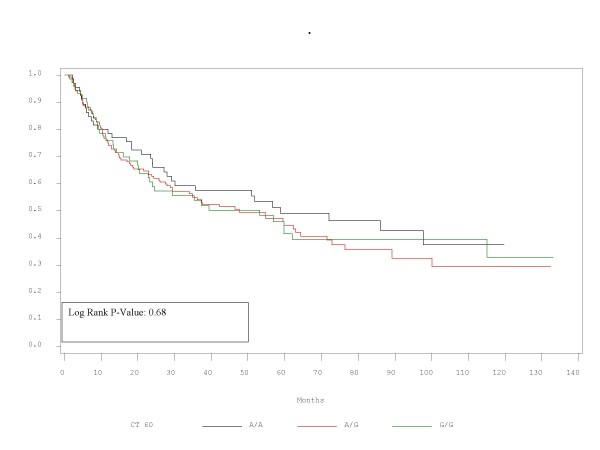
RFS plot by C/T 60 status

**Figure 6:**
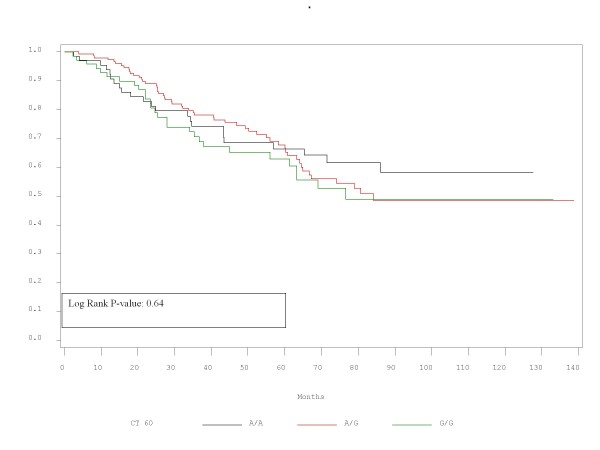
**OS plot by C/T 60 status**.

RFS and OS did not differ significantly between patients with the alleles represented by these polymorphisms. Τhe corresponding p-values for RFS and OS are presented in Table 2 and Figures 1,2,3,4,5,6. In addition, RFS and OS did not differ significantly in the cohort of patients with AG 49 GG when compared with patients with AG49 AA or AG (p = 0.5 and p = 0.51 respectively). No differences were again demonstrated when CT 318 CC and CT 60 GG where3 compared with the cohort of patients either heterozygous or homozygous to the protective allele (p = 0.38 and p = 0.58, and p = 0.92 and p = 0.38 respectively).

**Table 3:** CTLA-4 most frequent haplotypes

AG49	CT60	CT318	JO27	JO30	JO31	Chromosomes	
						
						Frequency (%)	Standard Error (%)
A	A	C	C	A	T	46.99	2.089
G	G	C	T	G	G	29.34	1.91
A	G	T	T	G	G	9.77	1.24
A	G	C	T	G	G	6.49	1.031
A	G	C	C	A	T	2.81	0.69

High association between the different polymorphisms was found (Fisher's exact p-value < 0.001 for all associations). Genotypes corresponding to the six CTLA-4 polymorphisms did not significantly deviate from the Hardy-Weinberg equilibrium. The test indicates significant linkage disequilibrium among the six polymorphisms

We analyzed the segregation pattern of CT 318, AG 49, CT 60, JO 27, JO 30, JO 31 SNPs on 572 chromosomes and identified 5 major haplotypes (table 3). No statistically significant differences for RFS or OS were found for the presence of each of the 3 most common haplotypes.

The association of Cw*06 with the CTLA-4 alleles was investigated and a statistically significant association was found with AG 49 (p = 0.023). In patients with positive Cw*06, 61.8% were AG 49 AA, 29.1% were AG 49 AG and 9.1% were AG 49 GG. The median relapse-free survival for Cw*06 positive patients with genotype AG 49 AG was 76.4 months and has not been reached yet for genotypes AA and GG. In Cw*06 negative patients the median relapse-free survival for genotypes AG49 AA, AG and GG was 56.7, 36.2 and 24.6 respectively. Median overall survival has not been reached yet in Cw*06 positive patients in all three genotypes (AG 49 AA, AG, GG) and in the Cw*06 negative cohort it was 86. 1, 66.7 and 61.2 months, respectively. However, no statistically significant differences were found for HLA Cw*06 positive patients in terms of RFS or OS, among AG 49 groups (p = 0.62 and p = 0.46 respectively). Likewise, no statistically significant differences were found for HLA Cw*06 negative patients in terms of RFS and OS among A/G 49 groups (p = 0.42 and p = 0.39 respectively). RFS and OS did not differ significantly in the cohort of patients with AG 49 GG vs AG/AA positive for HLA Cw*06 (p = 0.37 and p = 0.23 respectively) or negative for HLA Cw*06 (p = 0.22 and p = 0.22 respectively). In the cohort of patients included in the prospective autoimmune study, CTLA-4 polymorphisms were investigated in 157 out of 200 patients (48 autoimmunity group and 109 without evidence of autoimmunity). No statistically significant association was found among any of the six polymorphisms investigated. In the multivariate Cox model for RFS and OS, HLA Cw*06 and autoimmunity were statistically significantly correlated with RFS (p = 0.043 and p < 0.001 respectively), while only autoimmunity was found to be statistically significant for OS (p = 0.001).

## Discussion

This study analyzed the potential influences of the CTLA-4 genotype upon the outcome of IFN adjuvant therapy, on the basis of prior suggestions of the role of certain polymorphisms of the CTLA-4 gene and other immunotherapies for patients with melanoma. To answer these questions it was first necessary to define a baseline population for comparison. No database was available that describes the prevalence of CTLA-4 alleles among the Greek population, nor of melanoma patients from Greece. Several groups have reported analyses of the CTLA-4 genotypes of Caucasian and Japanese populations, yielding differing results [19,25,26]. Our results in the healthy Greek control population are similar to the allele frequencies identified in a population of 536 healthy Spanish haemopoietic stem cell donors that evaluated the association of CTLA-4 polymorphisms of patients and the post transplant outcome [27]. No significant differences were seen among the CTLA-4 profiles of the Greek healthy control and melanoma populations studied here.

Specific genetic polymorphisms of the CTLA-4 have been linked with an increased risk for multiple autoimmune diseases [17-19,25,26]. Intriguingly, a CTLA-4 polymorphism conferring low-level expression was found to be associated with higher frequencies of autoimmune toxicity among 19 melanoma patients treated concurrently with MDX-010 (ipilimumab) anti-CTLA-4 monoclonal antibody and a melanoma peptide vaccine [21]. An important result of this trial was the suggestion that the incidence of tumor relapse might be reduced among patients manifesting autoimmune toxicity. An earlier trial of concurrent MDX-010 and melanoma peptide vaccination also raised this possibility [28,29]. These provocative findings stimulated detailed investigation of the polymorphisms of CTLA-4 in larger numbers of patients treated in a subsequent study of 152 stage IV melanoma patients at the NIH. These investigators evaluated 7 common nucleotide polymorphisms and showed three SNPs to be associated with response to anti-CTLA4 antibody therapy: -1660AG, -657TC and AG 49. A haplotype analysis including the same 7 SNPs suggested that the common haplotype TACCGGG was associated with non-response (p = 0.02) whereas the haplotype TGCCAGG was associated with response to this treatment (p = 0.06). No significant association was observed among the occurrences of severe autoimmune reactions (grade III/IV) in patients with either single SNP or haplotype analyses [30].

The present cohort of patients with high risk melanoma has shown no correlation of any of the polymorphisms of CTLA-4 defined by the SNPs studied and improved RFS or OS, with adjuvant HDI treatment. Similarly, among 90 patients with stage IIB, IIC and III melanoma treated with HDI, AG 49 and CT 318 genotypes did not correlate with improved RFS and OS (Henry Koon, personal communication). There was a trend towards improved survival in the group with AG 49 AA (p = 0.06). The A allele of AG 49 was significantly associated with response (p = 0.009) among the 152 patients with stage IV melanoma treated with ipilimumab [30]. In the present study population, patients with the AG 49 AA allele had a better RFS and OS, but this did not reach statistical significance. This was also the case with the CT 60AA allele. The A allele at CT 60 has been identified as being responsible for a greater production of the soluble form of CTLA4 (s-CTLA4) [19,27], reflecting T-cell activation [31,32].

The GG allele was not associated with the development of autoimmunity in the cohort of patients retrospectively studied here. In the NIH Study [30] allele frequencies were also compared between groups of patients who developed autoimmune reaction of grade III/IV and those who did not--but no significant difference was observed. These findings may support the hypothesis that "induced autoimmunity" by IFN, IL-2, CTLA-4 blockade that is often a reversible process is a different process from spontaneous autoimmune disease. On the other hand, independent of genetic variation in CTLA-4, there was a strong positive association among response to the treatment and grade III/IV toxicity (p < 0.002) [30], as previously reported [28,29], and shown in our previously published work [15]. Failure to demonstrate the association of thyroid autoimmunity with certain CTLA-4 polymorphisms might indicate that the IFNa2b-related induction of autoimmunity in melanoma patients differs from spontaneously occurring autoimmune disorders with respect to the genetics of CTLA-4 and presumably, also in other aspects of this multi-factorial process. Thus, different routes to the development of autoimmunity may be associated with different sets of genes. Nevertheless, it is most interesting that a statistically significant association was found between HLA-Cw*06 and AG 49 allele distribution (p = 0.023). Although no statistical association was found between AG 49 alleles and RFS or OS, for HLA- Cw*06 positive patients, the ones with the GG genotype seemed to fair better regarding RFS and OS. Only one out of five patients had relapsed and all were alive. These results are limited by the small sample size and should be further explored in other trials of IFN-a2b of the US and European cooperative groups.

Our investigation into the association of CLTA-4 polymorphisms and the results of interferon therapy in a population where the occurrence of autoimmunity has been rigorously prospectively characterized, assumes that the predominant effect of CTLA-4 polymorphisms is upon T-cell responsiveness. The CT 60AA allele is associated with increased circulating levels of soluble CTLA-4, which adds another layer of complexity to these studies. Soluble CTLA-4 binds to CD80/86 and *in vitro *suppresses proliferation of committed autoreactive T cell clones in a dose-dependent manner [33]. However its function *in vivo *is unclear as s-CTLA-4 expression has been reported to correlate with the occurrence of autoimmunity. This dichotomy may reflect the fact that the effect of s-CTLA-4 is mediated by cells other that T-cells or that the expression of s-CTLA-4 during T-cell activation results in increased T-cell responsiveness, through inhibition of CTLA-4 ligation with CD80/86. Measurement of the pretreatment protein levels of s-CTLA-4 may give us more insight into the association between CTLA-4 polymorphisms and the clinical outcome of IFN therapy among patients receiving adjuvant interferon therapy or antibody mediated CTLA-4 blockade. These studies are now being planned.

## Conflicts of interest

Helen Gogas, Henry Koon, Michael Atkins and John Kirkwood has served as consultants to Schering Plough and have received honoraria from Schering Plough

## Authors' contributions

HG Conceived the study, participated in its design and coordination and helped to draft the manuscript, UD Participated in its design and performed the statistical analysis and helped to draft the manuscript, HK Conceived the study and helped to draft the manuscript, MSV Supervised the molecular genetics studies and is responsible for the quality control, YM Carried out the molecular genetic studies and participated in the sequence alignment, EB Carried out the molecular genetic studies and participated in the sequence alignment of the validating study. Collected and assembled the data, EP Carried out the molecular genetic studies and participated in the sequence alignment Collected and assembled the data, DT Provided study material.

AP Provided study material, AS Provided study material, CM Provided study material.

PP Provided study material, GF Provided study material and administrative support.

OC Provided study material, PS Participated in the sequence alignment, MBA Conceived the study and helped to draft the manuscript, JMK Conceived the study , participated in its design and helped to draft the manuscript.

All the authors read and approved the final manuscript.

## References

[B1] Kirkwood JM Strawderman MH Ernstoff MS Borden EC Blum RH Interferon alfa-2b adjuvant therapy of high-risk resected cutaneous melanoma: the Eastern Cooperative Oncology Group Trial EST 1684 J Clin Oncol 1996 14 7 17 8558223 10.1200/JCO.1996.14.1.7

[B2] Kirkwood JM Ibrahim JG Sosman JA Sondak VK Agarwala SS Ernstoff MS Rao U High-dose interferon alfa-2b significantly prolongs relapse-free and overall survival compared with the GM2 KLH/QS 21 vaccine in patients with resected stage IIB-III melanoma: results of intergroup trial E1694/S9512/C509801 J Clin Oncol 2001 19 2370 80 11331315 10.1200/JCO.2001.19.9.2370

[B3] Kirkwood JM Richards Th Zarour HM Sosman J Ernstoff M Whiteside TL Ibrahim J Blum R Wieand S Mascari R Immunomodulatory effects of high-dose and low-dose interferon a2b in patients with high-risk resected melanoma. The E2690 laboratory corollary of intergroup adjuvant trial E 1690 Cancer 2002 95 1101 1112 10.1002/cncr.10775 12209697

[B4] Yurkovetsky ZR Kirkwood JM Edington HD Marrangoni AM Velikokhatnaya L Winans MT Gorelik E Lokshin AE Multiplex analysis of serum cytokines in melanoma patients treated with interferon alpha2b Clin Cancer Res 2007 13 8 2422 8 10.1158/1078-0432.CCR-06-1805 17438101

[B5] Atkins MB Mier JW Parkinson DP Gould JA Berkman EM Kaplan MM Hypothyroidism after treatment with interleukin-2 and lymphokine-activated killer cells N Engl J Med 1988 318 1557 62 3259674 10.1056/NEJM198806163182401

[B6] Weijl NI Van Der Harst D Brand A Kooy Y Van Luxemburg S Schroder J Lentjes E Van Rood JJ Cleton FJ Osanto S Hypothyroidism during immunotherapy with interleukin-2 is associated with antithyroid antibodies and response to treatment J Clin Oncol 1993 11 1376 83 8315436 10.1200/JCO.1993.11.7.1376

[B7] Scalzo S Gengaro A Boccoli G Masciulli R Giannella G Salvo G Marolla P Carlini P Massimini G Holdener EE Primary hypothyroidism associated with interleukin-2 and interferon alpha-2 therapy of melanoma and renal carcinoma Eur J Cancer 1990 26 1152 6 10.1016/0277-5379(90)90275-X 2149997

[B8] Krouse RS Royal RE Heywood G Weintraub BD White DE Steinberg SM Rosenberg SA Schwartzentruber DJ Thyroid dysfunction in 281 patients with metastatic melanoma in renal carcinoma treated with interleukin-2 alone J Immunother Emphasis Tumor Immunol 1995 18 272 8 8680655 10.1097/00002371-199511000-00008

[B9] Phan GQ Attia P Steinbrg SM White DE Rosenberg SA Factors associated with response to high-dose interleukin-2 in patients with metastatic melanoma J Clin Oncol 2001 19 3477 82 11481353 10.1200/JCO.2001.19.15.3477

[B10] Becker JC Winkler B Klingert S Bröcker EB Antiphospholipid syndrome associated with immunotherapy for patients with melanoma Cancer 1994 73 1621 4 10.1002/1097-0142(19940315)73:6<1621::AID-CNCR2820730613>3.0.CO;2-E 8156489

[B11] Rosenberg SA White DE Vitiligo in patients with melanoma: normal tissue antigens can be targets for cancer immunotherapy J Immunother Emphasis Tumor Immunol 1996 19 81 4 8859727

[B12] Nordlund JJ Kirkwood JM Forget BM Milton G Albert DM Lerner AB Vitiligo in patients with metastatic melanoma: a good prognostic sign J Am Acad Dermatol 1983 9 689 96 10.1016/S0190-9622(83)70182-9 6643767

[B13] Bystryn JC Rigel D Friedman RJ Kopf A Prognostic significance of hypopigmentation in malignant melanoma Arch Dermatol 1987 123 1053 5 10.1001/archderm.123.8.1053 3631983

[B14] Schallreuter KU Levenig C Berger J Vitiligo and cutaneous melanoma. A case study Dermatologica 1991 183 239 45 10.1159/000247693 1809584

[B15] Gogas H Ioannovich J Dafni U Stavropoulou-Giokas C Frangia K Tsoutsos D Panagiotou P Polyzos A Papadopoulos O Stratigos A Markopoulos C Bafaloukos D Pectasides D Fountzilas G Kirkwood JM Prognostic significance of autoimmunity during treatment of melanoma with interferon N Engl J Med 2006 354 709 718 10.1056/NEJMoa053007 16481638

[B16] Dariavach P Mattei MG Golstein P Lefranc MP Human Ig superfamily CTLA-4 gene: chromosomal localization and identity of protein sequence between murine and human CTLA-4 cytoplasmic domains Eur J Immunol 1988 18 1901 1905 10.1002/eji.1830181206 3220103

[B17] Kristiansen OP Larsen ZM Pociot F CTLA-4 in autoimmune diseases--a general susceptibility gene to autoimmunity? Genes Immun1 2000 1 170 84 10.1038/sj.gene.6363655 11196709

[B18] Thompson CB Allison JP The emerging role of CTLA-4 as an immune attenuator Immunity 1997 7 4 445 50 10.1016/S1074-7613(00)80366-0 9354465

[B19] Ueda H Howsan JMM Esposito L Heward J Snook H Chamberlain G Rainbow DB Hunter KM Smith AN Di Genova G Herr MH Dahlman I Payne F Smyth D Lowe C Twells RC Howlett S Healy B Nutland S Rance HE Everett V Smink LJ Lam AC Cordell HJ Walker NM Bordin C Hulme J Motzo C Cucca F Hess JF Metzker ML Rogers J Gregory S Allahabadia A Nithiyananthan R Tuomilehto-Wolf E Tuomilehto J Bingley P Gillespie KM Undlien DE Rønningen KS Guja C Ionescu-Tîrgovişte C Savage DA Maxwell AP Carson DJ Patterson CC Franklyn JA Clayton DG Peterson LB Wicker LS Todd JA Gough SC Association of the T-cell regulatory gene CTLA-4 with susceptibility to autoimmune disease Nature 2003 423 506 511 10.1038/nature01621 12724780

[B20] Gough SCL Walker LSK Sansom DM CTLA-4 gene polymorphism and autoimmunity Immunological Reviews 2005 204 102 115 10.1111/j.0105-2896.2005.00249.x 15790353

[B21] Sanderson K Scotland R Lee P Liu D Groshen S Snively J Sian S Nichol G Davis T Keler T Yellin M Weber J Autoimmunity in a phase I trial of a fully human anti-cytotoxic t-lymphocyte antigen-4 monoclonal antibody with multiple melanoma peptides and montanide ISA 51 for patients with resected stages III and IV melanoma J Clin Oncol 2005 23 741 750 10.1200/JCO.2005.01.128 15613700

[B22] Gogas H Kirkwood JM Falk CS Sondak VK Tsoutsos D Stratigos A Markopoulos C Pectasides D Spyropoulou-Vlachou M Correlation of molecular human leukocyte antigen typing and outcome in high-risk melanoma patients receiving adjuvant interferon Cancer 2010 116 4326 33 10.1002/cncr.25211 20549830 PMC2970916

[B23] Pectasides D Dafni U Bafaloukos D Skarlos D Polyzos A Tsoutsos D Kalofonos H Fountzilas G Panagiotou P Kokkalis G Papadopoulos O Castana O Papadopoulos S Stavrinidis E Vourli G Ioannovich J Gogas H Randomized phase III study of 1 month versus 1 year of adjuvant high-dose interferon alfa-2b in patients, with resected high risk melanoma J Clin Oncol 2009 27 6 939 944 10.1200/JCO.2008.16.3121 19139440

[B24] Kaplan EL Meier P Non parametric estimation from incomplete observation J Am Stat Assoc 1958 53 457 481 10.2307/2281868

[B25] Furugaki K Shirasawa S Ishikawa N Ito K Ito K Kubota S Kuma K Tamai H Akamizu T Hiratani H Tanaka M Sasazuki T Association of the T-cell regulatory gene CTLA-4 with Graves' disease and autoimmune thyroid disease in the Japanese J Hum Genet 2004 49 166 168 10.1007/s10038-003-0120-5 14986169

[B26] Ikegami H Awata T Kawasaki E Kobayashi T Maruyama T Nakanishi K Shimada A Amemiya S Kawabata Y Kurihara S Tanaka S Kanazawa Y Mochizuki M Ogihara T The association of CTLA-4 polymorphism with type 1 diabetes is concentrated in patients complicated with autoimmune thyroid disease: a multicenter collaborative study in Japan J Clin Endocrinol Metab 2006 91 1087 1092 10.1210/jc.2005-1407 16352685

[B27] Perez-Gardcia A De la Camara R Roman-Gomez J CTLA-4 polymorphisms and clinical outcome after allogeneic stem cell transplantation from HLA-identical sibling donors Blood 2007 100 461 467 10.1182/blood-2007-01-069781 17384200

[B28] Phan GQ Yang JC Sherry RM Hwu P Topalian SL Schwartzentruber DJ Restifo NP Haworth LR Seipp CA Freezer LJ Morton KE Mavroukakis SA Duray PH Steinberg SM Allison JP Davis TA Rosenberg SA Cancer regression and autoimmunity induced by cytotoxic T lymphocyte-associate antigen 4 blockade in patients with metastatic melanoma Proc Natl Acad Sci USA 2003 100 8372 8377 10.1073/pnas.1533209100 12826605 PMC166236

[B29] Attia P Phan GQ Maker AV Robinson MR Quezado MM Yang JC Sherry RM Topalian SL Kammula US Royal RE Restifo NP Haworth LR Levy C Mavroukakis SA Nichol G Yellin MJ Rosenberg SA Autoimmunity correlates with tumor regression in patients with metastatic melanoma treated with anti-cytotoxic T-lymphocyte antigen-4 J Clin Oncol 2005 23 6043 6053 10.1200/JCO.2005.06.205 16087944 PMC1473965

[B30] Breunis WB Tarazona-Santos E Chen R Kiley M Rosenberg SA Chanock SJ Influence of Cytotoxic T lymphocyte-associated antigen 4 (CTLA4) common polymorphisms on outcome in treatment of melanoma patients with CTLA-4 blockade J Immunother 2008 31 586 590 10.1097/CJI.0b013e31817fd8f3 18528295 PMC3276400

[B31] Oaks MK Hallett KM Cutting edge: a soluble form of CTLA-4 in patients with autoimmune thyroid disease J Immunol 2000 164 5015 5018 10799854 10.4049/jimmunol.164.10.5015

[B32] Liu MF Wang CR Chen PC Fung LL Increased expression of soluble cytotoxic T-lymphocyte-associated antigen-4 molecule in patients with systemic lupus erythematosus Scan J Immunol 2003 57 568 572 10.1046/j.1365-3083.2003.01232.x 12791095

[B33] Huurman VA Unger WW Koeleman BP Oaks MK Chandraker AK Terpstra OT Roep BO Differential inhibition of autoreactive memory- and alloreactive naïve T cell responses by soluble cytotoxic T lymphocyte antigen 4 (sCTLA4), CTLA4Ig and LEA29Y Clin Exp Immunol 2007 150 487 93 10.1111/j.1365-2249.2007.03513.x 17924973 PMC2219382

